# Supplementary material for: Predicting Perceived Problems in Self-Administered 24-Hour Dietary Recalls: A Quantitative Think-Aloud Study Comparing Automated Self-Assisted 24-Hour Dietary Assessment Tool (ASA24®) and INTAKE24© in University Students
Source: Nutrients. 2022 Oct 13;14(20):4281. doi: 10.3390/nu14204281 (PMC9607278; doi:10.3390/nu14204281)
Supplement: Supplementary file 1 [file nutrients-14-04281-s001.zip › nutrients-1902567-supplementary.pdf]

Table S1. Creature of Habit Scale [32].

	**Routine Subscale**
**Habit_R_11**	I generally cook with the same spices/flavourings
**Habit_R_12**	I normally buy the same foods from the same grocery store
**Habit_R_15**	In a restaurant, I tend to order dishes that I am familiar with
**Habit_R_13**	I always follow a certain order when preparing a meal
**Habit_R_14**	I generally eat the same things for breakfast every day
	**Automaticity Subscale**
**Habit_A_5**	I often find myself finishing off a packet of biscuits just because it is lying there
**Habit_A_1**	I often find myself opening up the cabinet to take a snack
**Habit_A_10**	When walking past a plate of sweets of biscuits, I can't resist taking one
**Habit_A_7**	Television makes me particularly prone to uncontrolled eating
**Habit_A_2**	I often find myself eating without being aware of it
**Habit_A_11**	I am prone to eating more when I feel stressed
**Habit_A_9**	Eating crisps or biscuits straight out of the packet is typical of me
**Habit_A_6**	Whenever I go into the kitchen, I typically look in the fridge
**Habit_A_8**	I usually treat myself to a snack at the end of the workday
**Habit_A_3**	I often take a snack while on the go (e.g., when driving, walking, down the street, or surfing the web)

	**Removed items (not related to food consumption)**
	**Routine Subscale**
**Habit_R_1**	I tend to like routine
**Habit_R_2**	I find comfort in regularity
**Habit_R_3**	I rely on what is tried and tested rather than exploring something new
**Habit_R_4**	I quite happily work within my comfort zone rather than challenging myself
**Habit_R_5**	I tend to stick with the version of the software packages that I am familiar with for as long as I can
**Habit_R_6**	I tend to do things in the same order every morning (e.g., get up, go to the bathroom, have a coffee…)
**Habit_R_7**	I always try to get the same seat in places such as on the bus, in the cinema, or in church
**Habit_R_8**	I always like to park my car or bike always in the same place
**Habit_R_9**	I am one of those people who get really annoyed by last minute cancellations
**Habit_R_10**	I tend to go to bed at roughly the same time every night
**Habit_R_16**	I usually sit at the same place at the dinner table
	**Automaticity Subscale**
**Habit_A_4**	I often find myself running on autopilot, and then wonder why I ended up in a particular place or doing something that I did not intend to do

Table S2. Mindfulness Eating Questionnaire [23].

**Awareness Subscale**	
**Mind_A_1**	Before I eat, I take a moment to appreciate colours and smells of food
**Mind_A_2**	I taste every bite of food I eat
**Mind_A_3**	I appreciate the way my food looks on my plate
**Mind_A_4**	I notice when foods and drinks are too sweet
**Mind_A_5**	I notice subtle flavours in the food I eat
**Mind_A_6**	I notice when I am eating from a dish of candy just because it is there
**Mind_A_7**	I notice when the food I eat affects my emotional state
**Mind_A_8**	When I eat a big meal, I notice if it makes me feel heavy or sluggish
**Mind_A_9**	When eating a pleasant meal, I notice if it makes me feel relaxed
**Mind_A_10**	I recognise when food advertisements make me want to eat
**Mind_A_11**	I recognise when I am eating and not hungry
**Recognition Subscale**	
**Mind_R_1**	If there is good food at a party, I will continue eating even after I am full
**Mind_R_2**	if there are leftovers that I like, I take a second helping even though I am full
**Mind_R_3**	When I eat at ‘all you can eat buffets’, I tend to overeat
**Mind_R_4**	When I am eating one of my favourite foods, I do not recognise when I have had enough
**Mind_R_5**	If it does not cost much more, I get the larger size of food or drink, regardless of how hungry I am
**Mind_R_6**	I snack without noticing I am eating
**Mind_R_7**	At a party with a lot of good food, I notice when it makes me want to eat more than I should
**Mind_R_8**	When a restaurant portion is too large, I stop eating when I am full
**Mind_R_9**	I stop eating when I am full even when eating something I love

Problems with remembering	Tally
**What was eaten:**<br>*Participant may stumble, tut, say 'ummm' or question 'what did I eat' when inputting their item. E.g.:*<br>☐ Unsure what was eaten/ or if they ate<br>☐ Unsure if that's all they had to eat/ amount eaten<br>☐ Hesitation or long contemplation time on remembering what was eaten<br>☐ Prompt on the screen or task reminding them what they forgot	
**Problems with remembering**	**Tally**
**Item's portion:**<br>*Participant may stumble, tut, say 'ummm' or 'how much did I have' or 'what ingredients were in it'. Often associated with pre-bought/machine made or restaurant meals E.g.:*<br>☐ Unsure on ingredients of a meal<br>☐ Unsure of the portion due to machine, pre-packaged<br>☐ Unsure on portion due to meal made for more than one serve<br>☐ Correcting later or contradicting food item<br>☐ Unsure if/when they ate	
**Problems with remembering**	**Tally**
**Unsure on items specifically:**<br>*Participant may stumble, tut, say 'ummm' or 'what was it called' or 'what was the brand'. Often associated with pre-bought items E.g.:*<br>☐ Unsure where the item was from<br>☐ Unsure on the name or brand of the item<br>☐ Unsure of how it was commercially cooked	
**Problems with remembering**	**Tally**
**Guessing:**<br>*E.g.:*<br>☐ Guessing the time frame<br>☐ Guessing the amount eaten<br>☐ Guessing food due to missing item/Unsure on food item replacement	
**Problem with program**	**Tally**
**Inputting item into program: what is it?**<br>*Unsure what the program is asking from the participant. This can sound like confusion and is often associated with questioning themselves about what the program wants. E.g.:*<br>☐ Unsure when to search for an item<br>☐ Unsure where to search an item<br>☐ Unsure whether to add an item or not<br>☐ Unsure if program wanted it input separate or together<br>☐ Unsure what the program was asking (i.e., how much was left or how much drank)<br>☐ Unsure whether to put item as abbreviation or full name<br>☐ Unsure on the time of day recalling food from	

Figure S1. Perceived Problems Checklist.

Problem with program	Tally
**Inputting item into program: where is it?**<br>*Unsure where to find something on the program. Participants may speak aloud something like 'where is or what do I add it as...' and can be followed by incorrect item or amount being input. E.g.:*<br>☐ Can't find the food item on the program<br>☐ Unsure what to do when can't find item in the system<br>☐ Unsure what the food is called in the system<br>☐ Unsure what kind the item is categorised under	
**Problem with program**	**Tally**
**Individual problems:**<br>*E.g.:*<br>☐ Issues with correct spelling<br>☐ Exotic food, doesn't know the name/spelling<br>☐ Measurement of portion size is guessed or measured as more than on portion size<br>☐ Chose wrong day on the program	
**Emotional problems**	**Tally**
**Individual frustration:**<br>*A participant is sounding frustrated by the study. This is sometimes recognised as a large sigh, grunt or sometimes swear-word (if the context assumes frustration). E.g.:*<br>☐ Frustrated at the specificity of the program<br>☐ Frustrated putting every item in instead of an overall meal<br>☐ Frustrated at how long it's taking to recall everything or the length of the program	
**Emotional problems**	**Tally**
**Individual confusion:**<br>*A participant is sounding confused this can be when they are unaware of what the question asks or how to answer it. Voice generally heightens in pitch and questions themselves (not the program). E.g.:*<br>☐ Unsure what qualifies for mealtime of day<br>☐ Unsure what qualifies as a meal<br>☐ Unsure on why the program skipped and had to refill something in<br>☐ Struggles to find something on the program<br>☐ Confused with how to use the program<br>☐ Unsure on the unit of measurement	
**None**	**Tally**
**No problems arise**<br>*E.g.:*<br>☐ Participant chooses not to speak or has no problems arise in the study	

PROGRAM:

UNIQUE IDENTIFICATION CODE:

PARTICIPANT NUMBER:

Figure S1. *Continued.*

Instructions from S3. Within the left column are categories for perceived problems (Problems with remembering, problems with the program, emotion problems, and none) that provide descriptions and examples for what may be perceived as a problem during recall. Each time a problem in the category arose, a tally mark would go in the opposite box. All tallies should be summed for each category and subcategory. [Remembering (rows 1–4), Program (rows 5–7), Emotion (rows 8–9), None (row 10)].

Table S3. Systems Usability Scale [37].

**SUS_1**	I think that I would like to use this system frequently
**SUS_2R**	I found the system unnecessarily complex
**SUS_3**	I thought the system was easy to use
**SUS_4R**	I think that I would need the support of a technical person to be able to use this system
**SUS_5**	I found the various functions in the system were well integrated
**SUS_6R**	I thought there was too much inconsistency in the system
**SUS_7**	I would imagine that most people would learn to use this system very quickly
**SUS_8R**	I found the system very awkward to use
**SUS_9**	I felt very confident using the system
**SUS_10R**	I needed to learn a lot of things before I could get going with this system

Item 8′s wording of ‘cumbersome’ modified to ‘awkward’. Even items are reversed coded.
